# Application value and challenge of traditional Chinese medicine carried by ZIF‐8 in the therapy of ischemic stroke

**DOI:** 10.1002/ibra.12007

**Published:** 2021-12-11

**Authors:** Xiao‐Xi Zhu, Dong‐Fen Guo, Ming Chen, Xiao‐Qiong An, Bi Wang, Wen‐Feng Yu

**Affiliations:** ^1^ Key Laboratory of Molecular Biology Guizhou Medical University Guiyang Guizhou China; ^2^ Key Laboratory of Endemic and Minority Diseases, Education Ministry Guizhou Medical University Guiyang Guizhou China; ^3^ School of Basic Medical Science Guizhou Medical University Guiyang Guizhou China

**Keywords:** ischemic stroke, nanopreparation, traditional Chinese medicine, zeolitic imidazolate framework‐8

## Abstract

Stroke is a group of major diseases that cause death or disability in adults, with high incidence and lack of available therapeutic strategies. Although traditional Chinese medicine (TCM) has continuously achieved good effects in the therapy of stroke while there is still not convincing due to the limitation of blood–brain permeability, as well as the individual differences in usage and dosage. With the improvement of nanotechnology, TCM nanopreparation has gradually become a research hotspot in various fields due to its advantages in permeating the blood–brain barrier, targeting delivery, enhancing sustained‐release drug delivery, changing the distribution in the body, and improving bioavailability. Zeolitic imidazolate framework‐8 (ZIF‐8) is an ideal nano‐drug delivery system for adsorption, catalysis, and drug loading, which is a biocompatible metal–organic framework framed by 2‐methylimidazole and zinc ions. At present, ZIF‐8 was wildly used in the treatment of ischemic stroke. However, challenges remain persists for its clinical application, such as preparation technology, detection technology in vivo, targeting specificity, safety and stability, and so forth. Therefore, more efforts need to overcome the above problems to develop the application of TCM nanopreparations in the therapy of ischemia/reperfusion in the future.

## Graphical abstract

Research progress of the application of traditional Chinese medicine (TCM), TCM nanopreparation, and zeolitic imidazolate framework‐8 (ZIF‐8) nano‐drug delivery system in the treatment of ischemic stroke were reviewed in this article to explore the clinical application value and future challenges of ZIF‐8 as a nano‐drug delivery system in the treatment of nerve injury after ischemic stroke.

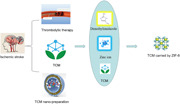

## 1 INTRODUCTION

Stroke, as the second leading cause of death in the world, is receiving more and more focusses due to the multiple serious threats to human health such as high morbidity, high disability rate, high mortality rate, and high recurrence rate.^1^ Meanwhile stroke gradually increased the global economic burden.^2^, ^3^ A total of 23%–65% of stroke patients with infectious complications resulted in unfavorable prognosis,^4^ and most of the surviving patients lose the ability to work to various degrees.^5^ Approximate 2.4 million new stroke cases are confirmed and appropriate 1.1 million people die from stroke each year in China, and the incidence of stroke tends to be younger.^6^ Statistically the incidence of cerebrovascular disease will increase by approximately 50% compared with that in 2010 in China by 2023.^7^

Stroke is mainly classified into ischemic stroke and hemorrhagic stroke with ischemic stroke incidence accounting for about 87% of all types of strokes. For prevention and treatment of stroke, institutions have respectively formulated and published multiple versions of the “Chinese Acute Ischemic Stroke Diagnosis and Treatment Guidelines,” including the Neurology Branch of the Chinese Medical Association, the Chinese Society of Traditional Chinese Medicine (TCM), and the Neurology Professional Committee of the Chinese Integrative Medicine Society,^8^ “Consensus on the Diagnosis and Treatment of Hemorrhage Transformation after Acute Cerebral Infarction in China 2019,”^9^ “Interpretation of Guidelines for Clinical Diagnosis and Treatment of Traditional Chinese Medicine”^10^ and “Diagnostic Standards of Integrated Traditional Chinese and Western Medicine for Cerebral Infarction and Cerebral Hemorrhage.”^11^ These guidelines and standards have strongly promoted the development of TCM and integrated traditional Chinese and western medicine diagnosis use in treatment of cerebral stroke in China and played a positive role in standardizing the diagnosis and treatment of stroke in China.

Thrombolytic therapy is the conventional clinical treatment of ischemic stroke whereas there is the risk of causing bleeding reperfusion injury, and some patients cannot receive effective treatment in time due to its short optimal treatment window. In recent years, proper results of TCM use in ischemic stroke have been proven successful in animal experiments. However, it is blocked by the blood–brain barrier (BBB), making most of the drugs unable to exert their effects. The modified nanomaterials developed an excellent brain drug delivery system due to the great advantage in penetrating BBB.^12^ TCM nanopreparations have gradually become a research hotspot in various fields due to their advantages in permeating BBB, targeting delivery, enhancing sustained‐release drug delivery, changing the distribution in the body, and improving bioavailability.

Metal–organic frameworks (MOFs) are ideal materials for adsorption, catalysis, and drug loading due to their large specific surface area, easy modification of the surface, high porosity, easy adjustment of pore size, structural diversity, many unsaturated metal‐binding sites, and good biocompatibility. Zeolite‐type imidazole ester framework materials (ZIFs) are subclusters of MOFs, which are topological nanomaterials formed by self‐assembly of transition metal atoms with organic imidazoles and their derivatives. Among them, zeolitic imidazolate framework‐8 (ZIF‐8) is a typical representative of ZIFs, and it is the first ZIFs material that have been found to have drug‐carrying functions. ZIFs have mild synthesis conditions, high pH sensitivity, close interaction with biomolecules, hydrothermal stability, and chemical stability. ZIF‐8 has the following advantages: high drug load, pH response characteristics, releasing high concentrations of drugs in certain acidic environments. ZIF‐8 can also be designed to respond to other stimuli type drug delivery systems (such as light, heat, magnetic field, positioning, GSH, H_2_O_2_ stimulation, etc.) control the release of drugs in specific parts and reduce the toxic and side effects on normal tissues; the shell of ZIF‐8 can protect the stability of biomolecules and improve biomolecules. The immobilized biomolecules are dispersed by ZIF‐8, which can avoid the aggregation of biomolecules, improve the activity of biomolecules and reduce the immunogenicity of biomolecules. ZIF‐8 has been extensively studied in the field of drug delivery due to its high porosity, ease of modification, and good biocompatibility. It has been widely used to assemble biomolecules by coprecipitation or bionic mineralization.^13^, ^14^ Currently, ZIF‐8 is wildly used as a drug delivery system for antitumor therapy, nerve injury treatment, and poststroke nerve injury treatment.

Research progress of the application of TCM, TCM nanopreparation, and ZIF‐8 nano‐drug delivery system in the treatment of ischemic stroke were reviewed in this article to explore the clinical application value and future challenges of ZIF‐8 as a nano‐drug delivery system in the treatment of nerve injury after ischemic stroke.

## 2 NERVE DAMAGE AND TREATMENT AFTER ISCHEMIC STROKE

### 2.1 Causes of nerve damage after ischemic stroke

After ischemic stroke, the normal transmembrane ion gradient and balance of neuron cells were broken, which leads to a series of cell death processes such as oxidative and nitrifying stress, excitotoxicity, inflammation, and apoptosis, resulting in neuronal cells apoptosis or necrosis.^15^, ^16^

### 2.2 Oxidative and nitrifying stress after ischemic stroke

Free radicals such as reactive oxygen species (ROS) and reactive nitrogen species may be important mediators of tissue damage in ischemic stroke. Free radicals formated by a variety of mechanisms mediated by N‐methyl‐D‐aspartic acid receptor (NMDA) receptors after variable types of stroke injury.^17^ Oxidative and nitrifying stress arises when there is an excess of free radicals over antioxidant defenses.^18^ Excessive ROS will damage cellular macromolecules then lead to tissue destruction and cell death through DNA oxidative damage, protein damage, intracellular calcium release, lipid peroxidation, cytoskeletal structure damage, and chemotaxis. Together with the oxidative stress caused by ROS, it affects the cerebrovascular system via altering the BBB's molecular structure and the expression of key tight junction proteins, damaging the BBB's functional integrity. In addition, ROS can significantly cause DNA damage and mitochondrial enzyme inhibition; and nitric oxide (NO) can also affect cell functions by regulating metalloproteinases, cysteine proteases, and glycolytic enzymes.^19^

### 2.3 Excitatory toxicity after ischemic stroke

Excitatory toxicity is one of the molecular mechanisms of ischemic injury. After stroke, glutamate, as a neurotoxic excitatory neurotransmitter, plays a key role in ischemia through excitotoxic mechanisms, resulting neuronal dysfunction and degeneration.^20^ Experiments have shown that Ca^2+^ is a key factor of glutamate neurotoxicity. Glutamate mainly acts through its receptors, among which the NMDA receptor is the most Ca^2+^ permeable glutamate ion receptor.^21^ Hyperactivation of NMDA receptors leads to excitotoxicity and neuronal death. Studies have shown that NMDA receptor blockers exert a neuroprotective effect by preventing Ca^2+^ from entering in vitro and vivo ischemic models.

### 2.4 Inflammation after ischemic stroke

After ischemic stroke, various molecules were activated and mediated the inflammatory cascade. The inflammatory process is related to several different cell types, inflammatory cytokines and cell receptors. The inflammatory response is initiated by the activation and recruitment of microglia. Then the microglia are induced to transform into phagocytes which produced various cytotoxic and/or cytoprotective substances. The original purpose of activated microglia is to protect neuronal cells, and microglia plays a neuroprotective role by releasing neurotrophic factors. However, excessive activation will produce proinflammatory cytokines in the ischemic area, such as interleukin 1β (IL‐1β), IL‐6, and tumor necrosis factor‐α (TNF‐α),^22^ as well as other potentially cytotoxic molecules, such as prostaglandins, ROS, NO, and so forth, resulting in harmful inflammation and neuronal apoptosis.^23^, ^24^ In addition to microglia involved in brain inflammation, neutrophils, monocytes, astrocytes, T lymphocytes, mononuclear phagocytes, natural killer cells, polymorphonuclear leukocytes, and other peripheral cells can also secrete cytokines then participate in ischemic inflammation.^25^, ^26^, ^27^

### 2.5 Apoptosis after ischemic stroke

Neuronal apoptosis is an important form of cell death. Two major pathways are divided, the death receptor (extrinsic) pathway, and mitochondria‐dependent (intrinsic) pathway. Studies have shown that inhibited neuronal apoptosis mitigates ischemic injury.^28^, ^29^

The endogenous pathway of cell apoptosis is realized on mitochondria, which is the center of energy synthesis and metabolism in the cell. While energy generation, it is also accompanied by the generation and release of ROS. When local brain tissue is ischemic, excessive production of ROS exceeds the ability of endogenous reductive substances to scavenge it. The accumulation of ROS in cells aroused oxidative stress, intracellular Ca^2+^ overload, toxins, ischemia, and hypoxia, resulting in the destruction of intracellular homeostasis, activation of the endogenous apoptosis pathway, finally induced cell apoptosis.

Ligand–receptor interaction initiating the exogenous cascade, which can induce the direct activation of caspase protein. Studies have reported that certain protective effects inhibited the activation of caspase protein on focal ischemic injury.^30^ FASL, TNF, LT‐α, LT‐β, CD40L, LIGHT, RANKL, TRAIL, and other TNF families bind to TNF receptors, Fas receptors (FasR), and TRAIL receptors, leading to the activation of death receptors and causing mitochondria membrane permeability, chromatin condensation, DNA fragmentation, and ultimately cell death.^31^

### 2.6 Treatment of ischemic stroke

The treatment of stroke is currently focused on how to improve blood flow restoration, cerebrovascular circulation, and neuroprotection. Shorten the time from consultation to recanalization, minimize the infarct size, and restore the ischemic brain tissue were the main strategy. Commonly used treatment regimens including thrombolytic therapy, intravascular interventional therapy, antithrombotic therapy, and neuroprotective were also based on these.

### 2.7 Thrombolytic therapy

Tissue‐type plasminogen activator therapy can effectively restore blood flow,^32^ while it is restricted by a narrow time window, contraindications, many complications, and a series of related risks.^33^, ^34^ As a result, only a small number of patients can receive thrombolytic therapy.^35^

### 2.8 Intravascular interventional therapy

Endovascular treatment methods include mechanical intravascular thrombus removal and arterial thrombolysis which improved clinical outcomes in patients with stroke.^36^, ^37^, ^38^ Because the therapeutic effect of arterial thrombolysis may be offset by delayed start‐up time, the clinical safety and effectiveness of angioplasty remain to be determined,^39^ mechanical intravascular thrombectomy is still the current first‐line endovascular treatment commonly used means.

### 2.9 Antithrombotic therapy

Studies demonstrated that the risk of recurrence of ischemic stroke decreased when combined aspirin and clopidogrel therapy within 12 h of onset,^40^ whereas it is not currently recommended for early anticoagulation therapy without selection.^41^

### 2.10 Neuroprotective therapy

The development and transformation of neuroprotective agents has gradually received more concentration in the therapy of ischemic stroke. Accordingly reported from domestic and foreign suggest that edaravone, a neuroprotective agent, improved the functional outcome of acute cerebral infarction. Administration of citicoline activated and improved brain metabolism and contribute to the recovery of brain function after brain injury,^42^, ^43^ but its efficacy needs to be further confirmed.^44^

For neuroprotective agents, it is difficult to control the therapeutic time window of drug treatment, the actual concentration in the ischemic brain tissue area, and individual differences in patients, resulting in poor clinical transformation effects. Therefore, finding effective drugs for the treatment of ischemic stroke is urgently required.^45^

Based on the many advantages of ZIF‐8 in drug delivery, corresponding therapeutic drugs can be codelivered according to the specific causes of nerve damage to improve the neuroprotective effect after ischemic stroke. For example, codelivery of tissue plasminogen activator for thrombolytic therapy, codelivery of aspirin and clopidogrel for antithrombotic therapy, codelivery of edaravone and citicoline for neuroprotective therapy, and so forth.

## 3 APPLICATION OF TCM IN THE TREATMENT OF ISCHEMIC STROKE

### 3.1 Overview of the prevention and treatment of ischemic stroke with TCM

“Stroke disease” which is characterized by sudden fainting, crooked tongue, hemiplegia, and poor language,^46^ weakness of blood circulation, and blood stasis. Clinically, TCM is used with conventional drugs for promoting blood circulation, removing blood stasis, and removing tendons and collaterals. Diagnosis and treatment of TCM has the characteristics of dynamic, syndrome differentiation and multicomponent, multitarget, and multipathway of TCM. TCM plays a major role in improving patients' neurological deficits, reducing inflammation, inhibiting cell apoptosis, and protecting nerve cells.

China National Stroke Database suggests that 70% of patients with ischemic stroke have been treated with Chinese medicine.^47^ A study in China involving 48 general hospitals indicated that 4.4% of patients with acute ischemic stroke had used Chinese medicine in 3 months before admission, and about 31.46% of patients continued to use proprietary Chinese medicines for treatment after discharge. Another prospective observational study suggested that the proportion of new‐onset ischemic stroke patients using Chinese medicine is as high as 83.1%, which has exceeded the use rate of antithrombotic drugs. According to a systematic review from 2015 to 2018, the combination of TCM is better than the conventional treatment of cerebral infarction in improving the neurological deficit of the patient with ischemic stroke within 2 weeks of onset.^48^

### 3.2 Research status of TCM in the treatment of ischemic stroke

In recent years, TCM has become more and more significant in the prevention and treatment of stroke due to its multiple targets, multiple pathways, and few side effects by continuous research and practice. TCM prescriptions, injections, and single TCM or its active ingredients, extracts are widely used in the study of neuroprotection after ischemic stroke (Tables 1, 2, 3). The therapeutic effect of stroke has been improved to varying degrees in different research fields.

**Table 1:** Research on the application of traditional Chinese medicine prescriptions in the prevention and treatment of ischemic stroke

Chinese herbal formula	Effect evaluation
Buyang Huanwu Decoction	It can improve patients' neurological deficits and spasm symptoms.^49^, ^50^, ^51^, ^52^, ^53^
Huangqi Guizhi Wuwu Decoction	It can improve the cerebral hemodynamics of patients, promote the recovery of nerve function, and has high safety.^54^, ^55^, ^56^, ^57^, ^58^
Taohong Siwu Decoction	It can inhibit cell coke death, improve the level of inflammatory factors, improve nerve function damage.^59^, ^60^, ^61^, ^62^, ^63^
Fuyuan Tongluo Decoction	It can improve limb motor function and nerve function, and can effectively reduce the sequela caused by cerebral infarction.^64^, ^65^, ^66^, ^67^
Huatuo Zaizao pills	It can significantly improve the neurological function, limb function, coagulation function, vascular endothelial function, and oxidative stress state of patients.^68^, ^69^, ^70^, ^71^, ^72^
Tongxinluo capsule	It can improve patients' cerebral hemodynamics, limb movement function, blood lipid, inflammatory factors, and improve clinical efficacy.^73^, ^74^, ^75^, ^76^, ^77^

**Table 2:** Study on the application of traditional Chinese medicine injections in the prevention and treatment of ischemic stroke

Traditional Chinese medicine injection	Effect evaluation
Ginkgolide injection	It can reduce the patient's inflammatory reaction, improve the patient's nerve function, and improve the ability of daily living.^78^, ^79^, ^80^, ^81^, ^82^
Danshen injection	It can improve the nerve function of patients, reduce the level of serum inflammatory factors, and improve the safety of patients' treatment.^83^, ^84^, ^85^, ^86^, ^87^
Danshen Ligustrazine injection	It can relieve the clinical symptoms and neurological dysfunction of patients and improve the clinical effect.^88^, ^89^, ^90^, ^91^
Shuxuetong injection	It can improve patient's nerve function and reduce adverse reactions.^92^, ^93^, ^94^, ^95^, ^96^
Xueshuantong injection	It can effectively promote neurological function, reduce inflammatory response, and reduce adverse reactions.^97^, ^98^, ^99^, ^100^, ^101^
Danhong injection	It can improve the patient's blood lipid level and brain function, with definite curative effect and high safety.^102^, ^103^, ^104^, ^105^, ^106^
Shuxuening injection	It can significantly improve neurological impairment, cognitive function, blood glucose index and inflammatory factor levels.^107^, ^108^, ^109^, ^110^, ^111^

**Table 3:** Study on the application of single Chinese medicine in the prevention and treatment of ischemic stroke

Single dose of Chinese medicine	Effect evaluation
Leech	It can improve the patient's nerve function and ability of daily living.^112^, ^113^, ^114^, ^115^, ^116^
Salvia	It can effectively reduce edema and improve neurological function.^117^, ^118^, ^119^, ^120^, ^121^
*Puerarin lobata*	It can significantly improve neurological function and has high safety.^122^, ^123^, ^124^
Ginkgo	It can reduce neurological damage and affect oxidative and inflammatory responses through multiple pathways.^88^, ^125^, ^126^, ^127^, ^128^
Astragalus	It has obvious protective effect on cerebral ischemia injury, and can treat ischemic stroke through multicomponent, multitarget, and multipathway.^129^, ^130^, ^131^, ^132^
Chuanxiong	It can inhibit inflammatory response, improve cerebral ischemia–reperfusion injury, protect nerve cells.^133^, ^134^
*Panax notoginseng*	It can improve the clinical efficacy of acute cerebral infarction and has good safety.^135^, ^136^

### 3.3 Problems in the prevention and treatment of ischemic stroke with TCM

There is still no unified guideline for the use of TCM in therapy of stroke due to the wide range of characteristics of TCM. Whether a single‐drug or a prescription for the treatment of ischemic stroke is largely dependent on the experience and subjective speculation of doctors. Therefore, the relevant molecular mechanism in the application of TCM needs further study. The working group of the Evidence‐Based Practice Guidelines for Stroke with Integrated Traditional Chinese and Western Medicine believe that in the future, high‐quality research should be given priority in the following areas: common clinical problems with high‐level evidence; re‐evaluation of classic TCM prescriptions with specific clinical positioning; whether the medicine is applicable or how to apply it to the links where the condition changes rapidly and the treatment risk is high.^137^

Although obvious potential advantages of TCM in the prevention and treatment of stroke, many problems need to be solved, for example, the complicated composition of TCM compound prescriptions, and the unclear mechanism; the reasonable compatibility of TCM in the compound requires in‐depth research; the long‐period used cycles and low‐efficiency; the recovery period and preventive treatment of stroke are prone to long‐term medication and the phenomenon of ignoring the occurrence of adverse reactions; the characteristics of low solubility, poor stability, and poor targeting of TCM components limit its clinical application.

### 3.4 Research status of TCM nanopreparations in the treatment of ischemic stroke

To expand the clinical application of TCM, more and more new drugs and routes of administration were investigated. With the development of nanomedicine and nanotechnology, nanomedicine has gradually become a research hotspot in the treatment of various diseases due to the following unique advantages. First, nanomedicine can increase the solubility of insoluble drugs, improve stability, and extend the half‐life of drugs in the body. Second, targeted modified nanomedicine can assist the drug to cross the BBR or be absorbed by specific cells (such as injured neurons), good therapeutic effect with the low dose and toxicity; finally, the drug can be controlled due to the different functional materials of nanomedicine.

Currently, liposomes, micelles, dendrimers, nanoparticles, and exosomes and other nanodrugs have been used in preclinical studies of ischemic stroke,^138^ and improved the ischemic brain through different ways, for example, improving the efficacy and safety of thrombolytic drugs,^139^, ^140^ delivering oxygen to the ischemic brain,^141^ regulating ion imbalance and excitotoxicity,^142^ reducing oxidative stress, reducing cell apoptosis, regulating inflammation and immune response,^143^ promoting tissue repair,^144^ regulating a variety of abnormalities,^145^ controlling the response and release of drugs at pathological sites.^146^, ^147^

In recent years, the TCM nanopreparations produced by the combination of TCM and nanotechnology have to some extent made up for the shortcomings of TCM such as long treatment course, slow response, low solubility of TCM ingredients, poor stability, and poor targeting. It also reduces drug toxicity, side effects, and dosage. Various nanoformulations of TCM have improved the therapeutic effect of ischemic stroke to varying degrees through different routes of administration (oral, injection, nasal cavity, inner ear, skin; Table 4).

**Table 4:** Research on the application of traditional Chinese medicine nanopreparations in the prevention and treatment of ischemic stroke

Nanopreparations	Delivery system	Effect evaluation
Naotong nasal drops	Nasal spray microemulsion	It can effectively protect the nerve function and promote the recovery of nerve function.^148^
Salvianolic acid B, tanshinone ⅡA	PLGA nanoparticles	It has a potential protective effect on cerebrovascular injury.^149^
Curcumin	Solid lipid nano	It can improve the availability and brain distribution of curcumin.^150^
Salvianolic acid B	Chitosan	It has good protective ability and can play a protective role in central nervous system diseases.^151^
Ginkgolide B	Polyethylene glycol	It can prolong the retention time of ginkgolide B in vivo and improve its bioavailability.^152^
*Panax notoginseng* saponins	Nasal powder mist	It has an obvious protective effect, and the higher the dose, the stronger the protective effect.^153^
Ganoderma triterpenes	Nanosuspension gel	It can increase skin surface bioavailability and reduce adverse drug reactions.^154^, ^155^
Tanshinone IIA	Polyethylene glycol	It can interfere with the occurrence of inflammatory response after stroke.^156^
Puerarin	Polybutylcyanoacrylate nanoparticles	It can increase the relative bioavailability of puerarin and enhance the protective effect on focal cerebral ischemia–reperfusion injury.^157^
Ginsenoside Rg1	Polyglutamic acid nano‐drug delivery system	It can cross the blood–brain barrier, promote the proliferation of blood vessels and nerve cells, reduce nerve cell apoptosis.^158^
Valerenic acid	Polyethylene glycol–polylactic acid	It can regulate inflammation and oxidative stress, inhibit cell apoptosis, and play a neuroprotective role.^159^

Abbreviation: PLGA, poly (lactic‐co‐glycolic acid).

However, there are also some problems in TCM nanopreparations, such as low drug load; low stability of the combination of nanocarriers and targeted drugs; the low tissue specificity targeted drugs; difficult to detect in vivo; the high cost of TCM nanopreparations. Therefore, overcoming the above problems is of great significance to the development of nanoformulations of TCM in the future.

### 3.5 Application and challenge of ZIF‐8 in neuroprotection

In recent years, based on many features and advantages of ZIF‐8, researchers have continuously used ZIF‐8 to assemble functional proteins, enzymes, nucleic acids, viruses, and gene‐edited originals for clinical anticancer treatment research,^160^, ^161^ all of which have improved tumor suppression effects to varying degrees. Based on ZIF‐8 as a nanocarrier to cooperate with one or more conventional tumor treatment methods (such as chemotherapy, phototherapy, chemotherapeutic therapy, starvation therapy, immunotherapy).

**Figure 1:**
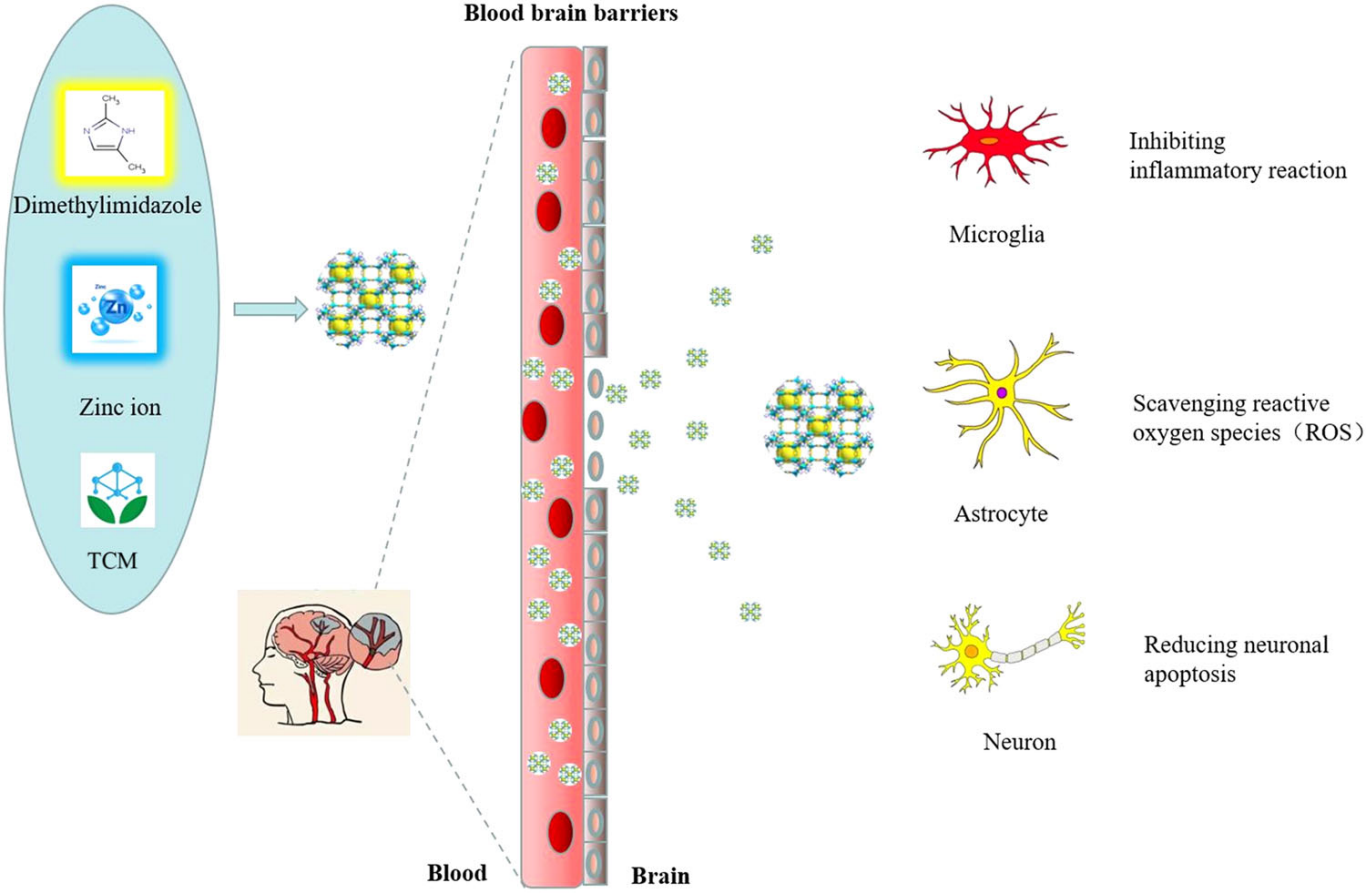
Schematic diagram of ZIF‐8 combined with TCM in the treatment of nerve injury caused by reperfusion of ischemic stroke. TCM, traditional Chinese medicine; ZIF‐8, zeolitic imidazolate framework‐8 [Color figure can be viewed at wileyonlinelibrary.com]

Also based on the many characteristics and advantages of ZIF‐8, many researchers have also applied ZIF‐8 in the treatment of nerve injury. Li et al.^162^ used biomimetic mineralization to construct nanoassembly SOD@ZIF‐8 for the treatment of Parkinson's disease (PD). The results showed that SOD@ZIF‐8 can effectively eliminate ROS in cells and organisms by alleviating oxidative damage to achieve the therapeutic effect on PD. Qin et al.^163^ prepared ZIF/Fer with ferrocene encapsulated ZIF‐8 and applied it to the study of AβO detection. The dual detection technology combining optical sensing and electrochemical analysis of two sensing methods has played a synergistic effect in the qualitative and quantitative detection of AβO, suggesting that ZIF/Fer is a promising AβO in vivo monitoring and analysis platform. He et al.^164^ designed ZIF‐8‐coated CeO_2_ nanoparticles (CeO2@ZIF‐8NPs) to enhance the catalytic and antioxidant activities of composite nanoparticles. The nanosystem can effectively inhibit middle cerebral artery occlusion mice. Lipid peroxidation in brain tissue reduces oxidative damage and apoptosis of neurons in brain tissue. CeO2@ZIF‐8‐attenuated injury by suppressed inflammation and immune responses via inhibiting the activation of astrocytes and the secretion of proinflammatory cytokines, thus exerting neuroprotective effects in the treatment of ischemic stroke. In situ synthesis pathway of synergistic nanotherapy combined with ZIF‐8 and CeO_2_ provide new ideas for the neuroprotection of ischemic stroke and reperfusion injury, prompting the use of ZIF‐8. The drug‐carrying system cocarrying TCM to treat nerve injury caused by ischemic stroke and reperfusion has potential application value (Figure 1).

Although many advantages of ZIF‐8 as a drug delivery system, there is insufficient evidence of clinical application. The mechanism of action is not completely clear, and there is insufficient evidence whether it can accurately treat damaged tissues and play a protective role. Because of TCM compound, many medicinal flavors, complex ingredients, and difficulty in in vivo pharmacokinetic research have led to the fact that most of the Chinese medicine nanopreparations currently studied are Chinese medicine monomers, and the research on Chinese medicine compounds and effective parts is relatively scarce. Given the diversity and complexity of nerve injury after ischemic stroke, it may be difficult to achieve the desired effect with a single agent. Therefore, the clinical application of ZIF‐8 still faces many challenges, such as nanopreparation technology, TCM compound coloading technology, detection technology in vivo, targeting specificity, safety and stability, and so forth. Overcoming of the above problems is of great significance in the development of TCM nanopreparations in the future

## 4 CONCLUSION

Although TCM and TCM nanopreparations have made some progress in neuroprotection in stroke, there are still many problems that need to be solved urgently. For example, the animal model used in the research is restricted, and the patient's clinical classification and phenotypic mismatch. Attention should be paid to the research of multitarget brain‐protective drugs, not only to protect neurons, but also to protect glial cells, blood vessels, and so forth. Although the nanoadministration route can pass through BBB, it cannot be confirmed whether it has reached the drug site. The mechanism of action of drugs in the brain is not clear. Nanodrugs are mostly in the laboratory research stage, and the effects of animal experiments and human experiments may be different, and there is still a lack of clinical data to prove that it is safe to apply in the clinic.

In view of the complexity of ischemic stroke injury, combination therapy may be more effective than a single drug. Compared with TCM monomers, compound medicines show more advantages. The synergistic effect of compound medicines through reasonable compatibility may have higher efficacy in neuroprotection after stroke. In the future, the research and development of nanopreparations of TCM can start from the compound prescriptions with less medicinal taste, clear active ingredients, significant therapeutic effects, and controllable quality, and carry them out with a nano‐drug carrier system with good biocompatibility, targeting, and safetyprocess of drug delivery in the organism in the, to achieve better development in the neuroprotection of ischemic stroke.

## CONFLICT OF INTERESTS

We declare that our research was conducted by ourself without copy any papers and there is no conflict of interest.

## ETHICS STATEMENT

Ethics statement is not applicable to this study.

## AUTHOR CONTRIBUTIONS

Xiao‐Xi Zhu and Dong‐Fen Guo designed the study. Ming Chen and Xiao‐Qiong An performed the experiments. Xiao‐Xi Zhu drafted the manuscript. Bi Wang contributed to the analysis of data and designed the animal experiment. Wen‐Feng Yu contributed to the conceptualization and methodology. All authors read and approved the final manuscript.

## Data Availability

The datasets used and/or analyzed during the current study are available from the corresponding author on reasonable request.
